# Energy Drinks and Arrhythmias in Adolescents and Young Adults: A Narrative Review of the Last Decade

**DOI:** 10.3390/medicina62081533

**Published:** 2026-08-09

**Authors:** Antonios Constantinou, Christos Kogias, Elpida Emmanouilidou-Fotoulaki, Eleni P. Kotanidou, Andreas Giannopoulos, Charalampos Antachopoulos, Assimina Galli-Tsinopoulou, Maria Kavga

**Affiliations:** 1 3rd Department of Pediatrics, School of Medicine, Aristotle University of Thessaloniki, 54124 Thessaloniki, Greece; constantinou.antonis@yahoo.com (A.C.); chrikogi@auth.gr (C.K.); bantacho@yahoo.gr (C.A.); 2 Postgraduate Program in Adolescent Medicine and Adolescent Health Care, School of Medicine, Faculty of Health Sciences, Aristotle University of Thessaloniki, 54124 Thessaloniki, Greece; epkotanidou@auth.gr (E.P.K.); agalli@auth.gr (A.G.-T.); 3 1st Department of Pediatrics, School of Medicine, Aristotle University of Thessaloniki, 54124 Thessaloniki, Greece; eemma@auth.gr; 4 2nd Department of Pediatrics, School of Medicine, Aristotle University of Thessaloniki, 54124 Thessaloniki, Greece; agianop@auth.gr

**Keywords:** energy drinks, arrhythmias, adolescents, young adults, ECG, literature review

## Abstract

*Background and Objectives*: Energy drinks (EDs), widely consumed by adolescents and young adults, contain caffeine and other stimulants marketed to enhance energy and performance. ED consumption has been associated with adverse cardiovascular effects, although most evidence derives from adult populations. This review examines the association between ED intake and arrhythmias or electrocardiographic (ECG) alterations in young people, including the influence of consumption patterns and the reversibility of reported changes. *Materials and Methods*: A review of the literature of the last ten years was conducted in the PubMed, Scopus, Cochrane Library and Google Scholar databases, using various combinations of the key words: “Energy Drinks”, “arrhythmias”, “ECG”, “electrocardiogram”, “dysrhythmias”, “children”, “teen”, “teenagers”, “adolescents”, “young adults”, “young people”. *Results*: 22 studies met the inclusion criteria: 10 RCTs, 5 non RCTs, 1 observational study and 6 case reports. Evidence suggests that ED consumption may be associated with cardiac rhythm disturbances and ECG alterations in young individuals. Reported events include atrial fibrillation, ventricular fibrillation, and, in rare cases, cardiac arrest, often following moderate-to-high ED intake. Experimental studies have demonstrated acute cardiovascular effects, including QTc interval changes (6–20 ms), increased supraventricular ectopic activity, alterations in heart rate (3–10 bpm) and ST–T changes. Most reported alterations appeared acute and reversible; however, their occurrence may be influenced by ED dose, composition, caffeine exposure, and individual susceptibility. *Conclusions*: ED consumption among adolescents and young adults may increase the risk of arrhythmias. There is consistent evidence across studies showing that the most common effects are HR and QTc alterations, with the composition and quantity of the drinks likely influencing these effects. Important gaps remain regarding frequent consumption, long-term outcomes, the contribution of less-studied ingredients, and the impact on younger populations. Stronger regulations may also be warranted to protect young consumers, particularly those at increased cardiovascular risk.

## 1. Introduction

Energy drinks (EDs) are beverages having high quantities of caffeine as their main ingredient, stimulants and sweeteners, which offer high energy, improved performance, attention, concentration, alertness and reduced fatigue to their consumers. They became popular during the 1980s in Europe and during the 1990s in the USA and since then they have gained immense commercial success, as more and more people tend to consume the numerous new drinks appearing in the market every year [1]. Their consumption is particularly popular among young people, who are also the main target of advertisements that appear frequently on platforms they often use [2]. According to a recent meta-analysis, the prevalence of ED consumption is 62.1% in North America and 55.5% in Europe. In terms of age groups, adolescents and young adults clearly show a superiority in consumption compared to children and adults, since >50% of these age groups have consumed an ED at least once in their life, >40% in the last year and >30% in the last month [3]. The main reasons why adolescents and young adults consume EDs according to questionnaire-based studies conducted in educational institutions around the world include: the desire for increased energy, the desire to stay awake, improving athletic performance, improving concentration, improving academic performance, the pleasant taste of the drinks, fun, curiosity and combining them with alcohol or other substances [4,5,6,7,8,9,10,11,12,13,14,15,16,17].

The literature shows a correlation between the consumption of EDs and adverse effects across multiple organ systems. These include neurological and psychiatric symptoms such as headache, dizziness, irritability, anxiety, psychotic episodes, tremors, and convulsions; gastrointestinal issues like abdominal pain, nausea, gastritis, hepatitis, pancreatitis; and urinary complications, notably acute kidney damage. Additionally, ED consumption has been linked to reduced duration and poor quality of sleep, reduced academic performance, increased risk of obesity, insulin resistance and metabolic syndrome. Reports also highlight allergic reactions, gynecological issues such as menorrhagia, and dermatological manifestations like rashes [18,19,20,21,22].

Regarding the cardiovascular system, palpitations, chest pain and hypertension are frequently reported following ED consumption. More serious events have also been described, including supraventricular and ventricular arrhythmias, syncope, and cardiac arrest. In addition, cases of coronary thrombosis, myocardial infarction, coronary vasospasm, aortic dissection, and cardiomyopathy have been reported in association with ED intake [18,19,20,21,22]. Notably, between 2012 and 2014, the United States Food and Drug Administration (FDA) recorded 34 deaths reported in association with energy drink consumption [23].

Given the widespread use of EDs among adolescents and young adults and the limited data on their cardiovascular safety in this population, this review aims to examine the association between EDs consumption and cardiac arrhythmias or electrocardiographic (ECG) alterations in young people, in which the anticipated prevalence of heart disease is low. It also explores whether these effects are related to the type, amount, and duration of consumption, and whether the reported arrhythmias or ECG changes are transient or reversible.

## 2. Materials and Methods

### Search Process

A comprehensive literature search was conducted in PubMed, the Cochrane Library, Scopus, and Google Scholar using the keywords “energy drink,” “arrhythmia,” “dysrhythmia,” “heart,” “ECG,” “electrocardiogram,” “children,” “teen,” “teenager,” “adolescent,” “young people,” and “young adult,” combined with Boolean operators (“AND,” “OR”). Articles published between January 2015 and April 2025 were considered if they involved participants aged 10–24 years (based on mean age), were written in English, and included randomized or non-randomized clinical studies, observational studies (cross-sectional, case–control, or cohort studies), case series, case reports, in which cardiac arrhythmias and/or electrocardiographic abnormalities were clearly described. Studies published before 2015, those involving participants younger than 10 or older than 24 years, non-English publications, reviews, animal or in vitro studies, letters to the editor, opinion or commentary articles, and articles not reporting confirmed arrhythmias or electrocardiographic alterations were excluded. Studies were selected independently by two reviewers who used a structured search strategy and inclusion/exclusion criteria in order to identify all eligible studies, while a senior expert resolved any possible disagreements. From the included publications, relevant data were extracted narratively and, where appropriate, tabulated, including authorship, year of publication, country, sample size, sex distribution, mean age, reported medical history, study design, characteristics of energy drink exposure (type, quantity, and duration of consumption, when available), and reported outcomes, specifically the occurrence and type of arrhythmias and electrocardiographic changes and whether these were transient or persistent. For case reports and case series, additional information regarding clinical presentation, management, and outcomes was also recorded. As an evaluation metric tool, SANRA (Scale for the Assessment of Narrative Review Articles) was used to ensure the methodological rigor and quality of the present review. The SANRA tool evaluates six domains: justification of article importance, statement of aims, literature search description, referencing, scientific reasoning, and appropriate presentation of data, with each domain scored on a 0 (low standard) to 2 (high standard) scale (maximum possible score: 12) (Appendix A). Two independent reviewers assessed the manuscript using SANRA—any discrepancies in scoring were resolved through discussion until a consensus was reached (Table A1) [24].

## 3. Results

A total of 318 records were identified through the literature search. After removal of duplicates and screening of titles and abstracts, 85 articles were assessed for eligibility. Two articles could not be retrieved despite attempts to contact the authors. Of the 83 full-text articles evaluated, 61 were excluded: 24 due to participant age > 24 years, 2 because the full text was not available in English, and 35 due to the absence of confirmed arrhythmias or electrocardiographic abnormalities. Ultimately, 22 studies met the inclusion criteria and were included in the review.

Of the selected articles, 15 refer to clinical studies—10 Randomized Controlled Trials (RCTs) [25,26,27,28,29,30,31,32,33,34] and 5 non RCTs [35,36,37,38,39], 1 refers to an observational study [40], 6 include case reports [41,42,43,44,45,46]. The characteristics of all the included studies are presented in Table 1, Table 2, Table 3 and Table 4.

Table 1 shows the findings of 10 RCTs including a total of 352 participants. The reviewed studies demonstrate that acute ED consumption is associated with measurable cardiovascular effects in healthy children, adolescents, and young adults.

Table 2 presents the findings of 5 non-RCTs including a total of 201 participants. In line with the RCTs mentioned before, the included non-randomized studies also indicate that acute ED consumption is associated with alterations in cardiovascular autonomic regulation, cardiac electrophysiology, and hemodynamic parameters, although the direction and magnitude of these effects vary across populations.

Table 3 shows the findings of a cross-sectional study [40], based on questionnaires investigating the presence of arrhythmias and the existence of proarrhythmogenic risk factors among 186 individuals.

Table 4 (case reports) summarizes 8 cases of individuals with major arrhythmias after ED intake either alone (quantities exceed 500 mL) [42,43,44,45] or combined with alcohol [41] or illicit substances [46]. Further evaluation of individuals without preexisting medical conditions, including serum electrolyte assessment, thyroid function testing, echocardiography, cardiac magnetic resonance imaging, coronary angiography, and genetic testing, was unremarkable.

### 3.1. ED and Heart Rate Changes

Several studies evaluated the effects of ED consumption on heart rate (HR), with heterogeneous findings. Some studies showed a significant HR increase (3.5–10 bpm) shortly after ED intake, particularly within 30–60 min, suggesting enhanced sympathetic activity [25,27,29,33,37,38]. In contrast, other investigations reported transient HR reductions (3–8 bpm) after ED consumption [31,32,34,36,39]. In the EDUCATE study, the mean HR difference between ED and placebo was −2.71 bpm, 60–120 min after their consumption [31]. These decreases were sometimes accompanied by increases in blood pressure, suggesting a possible reflex autonomic response [31,32]. Overall, ED intake appears to induce acute HR alterations, although the direction and magnitude of these changes vary across populations and experimental conditions.

### 3.2. ED and QTc Interval Changes

Multiple studies examined the impact of ED consumption on ECG parameters, particularly the QTc interval. Among the studies that specified the correction method, Bazett’s formula was used to calculate the QTc interval [26,30,31,35,38]. Several RCTs reported significant QTc prolongation following high-volume ED intake compared with placebo beverages [26,29,30] while others report a QTc shortening [27,29,38], suggesting potential alterations in cardiac electrophysiology. Other studies observed QTc changes influenced by individual characteristics, such as obesity or habitual caffeine consumption [35,38]. QTc changes ranged from approximately 6 to 20 ms—however QTc duration remained within normal limits. Overall, ED consumption may influence cardiac repolarization in susceptible individuals.

### 3.3. ED and Arrhythmias

Case studies [41,42,43,44,45,46] associated the consumption of ED alone or in combination with alcohol or illicit substances with severe arrhythmias such as VT and AF, in individuals without significant medical history (except in one case [42]).

### 3.4. ED and Other ECG Changes

Increased supraventricular ectopic activity was observed in children and adolescents after ED intake compared to placebo (IRR = 1.7) [31]. In addition, one study mentions more frequent ST-T alterations [36]. Some studies found no significant ECG alterations after ED consumption [28].

### 3.5. Duration—Transient or Persistent Alterations

Reported arrhythmias and electrocardiographic alterations were generally transient; however, participant monitoring in clinical studies did not exceed 12 h, and the effects of repeated or long-term consumption were not assessed, as all intervention studies evaluated only single-dose exposure. Therefore, whether these effects persist, accumulate, or differ with regular consumption remains unknown [26,27,29,30,31,32,33,34,35,36,37,38,39].

## 4. Discussion

All available published data from the past decade as reflected on this study indicated that ED consumption, either alone or in combination with alcohol or other substances, has been associated with heart rate alterations, ECG changes and more rarely with arrhythmias in young adults. Electrocardiographic alterations reported in clinical studies include changes in HR (both increases and decreases), QTc interval shortening or prolongation, ST–T segment changes, and an increased frequency of supraventricular extrasystoles. None of the interventional studies included in this review documented the occurrence of arrhythmias during monitored ED consumption. Top of Form Notably, among the identified studies [25,26,27,29,30,33,35,36,37,38,39], only one focused exclusively on children and adolescents (the EDUCATE study) [31,32,34,47].

**Figure 1:**
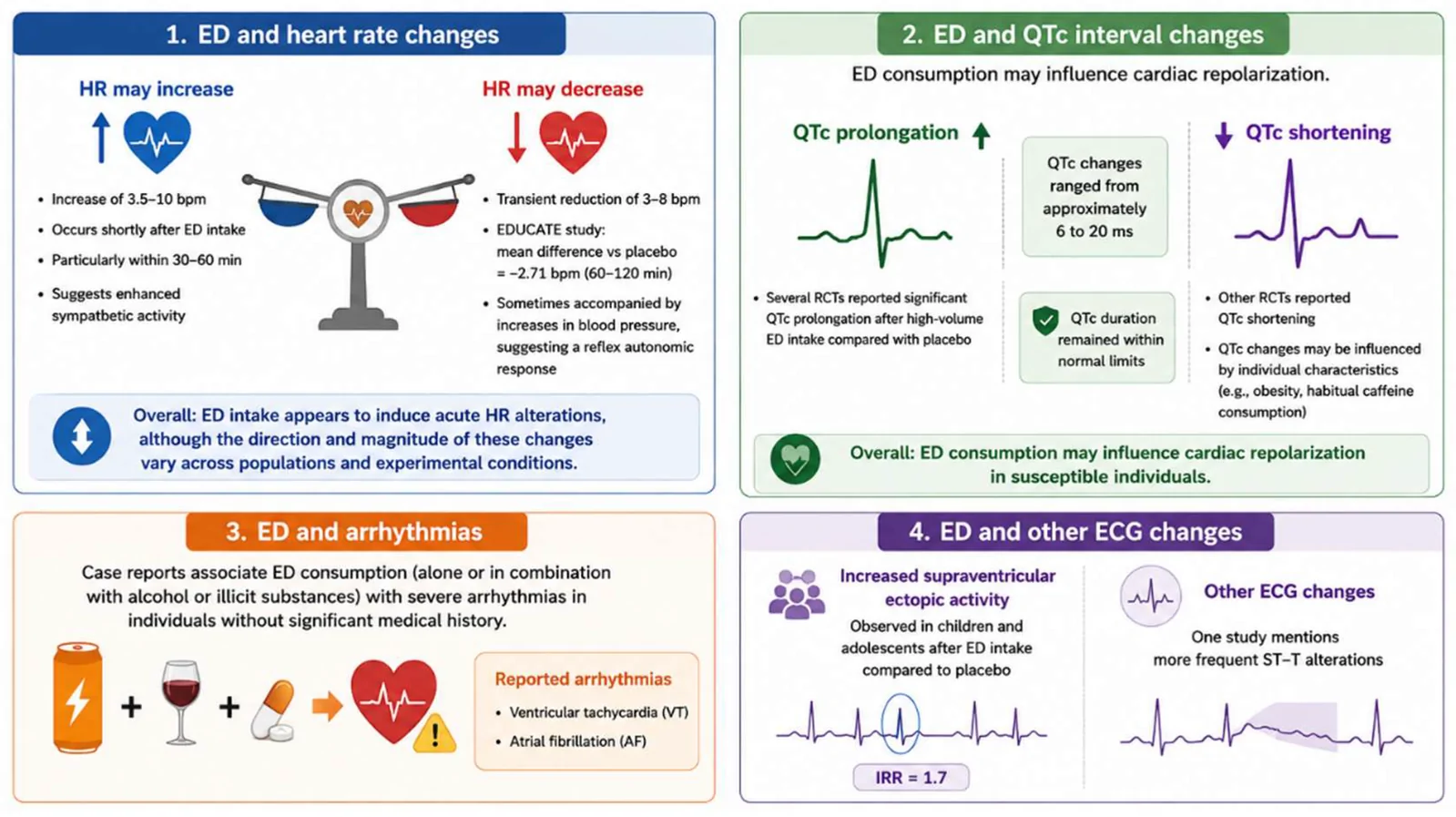
The effects of EDs on cardiac electrical activity.

Figure 1 summarizes the effects of EDs on cardiac electrical activity.

The present review initially sought to identify evidence from the past decade regarding an association between ED consumption and the occurrence of arrhythmias or electrocardiographic alterations in children and adolescents. However, due to the limited number of studies focusing exclusively on this population, the inclusion criteria were expanded to encompass individuals up to 24 years of age. This approach was adopted in accordance with the WHO definition of “young people” (10–24 years) [48], thereby allowing the inclusion of a broader body of relevant evidence. In the last decade, only a few reviews studying the effects of EDs referred exclusively to children, adolescents and young people [20,21] while others had no age limitation [18,19,22]. Although they mentioned possible adverse effects on the cardiovascular system, these studies did not provide a detailed description of the types of arrhythmias or the specific electrocardiographic alterations that may occur.

Overall body of evidence supports the effects of ED consumption on cardiovascular system as indicated through the controlled trials included in this review. With only one study finding no association [28], the majority reported effects in the same direction. The consistency of these findings across RCTs, where confounding factors are controlled by design, strengthens the observed association. Some of their conflicting results could be attributed to the different ED used, the different amount administered, the different follow-up time, and differences in participant characteristics.

Changes in HR were reported in nine of the included studies. Most studies observed an increase in HR [27,29,33,37,38,49] whereas three studies, including the only one conducted in adolescents and children, reported a reduction in HR [31,36,39]. These alterations were predominantly observed 60–120 min after ED consumption, corresponding to the time at which plasma caffeine concentrations typically peak [50]. In studies reporting an increase in HR, this effect was primarily attributed to caffeine-mediated antagonism of A1 and A2 adenosine receptors, leading to increased catecholamine release. However, the contribution of other ED constituents cannot be excluded. For instance, Caliskan et al. [37] reported a significant increase in HR following ED consumption but not after the intake of other caffeinated beverages, suggesting a potential role of additional ingredients. Across studies, the reported HR increase ranged from +3.5 to 10 bpm, reaching up to 20 bpm following exposure to mental stress. As noted by Grasser et al. [25], this response appeared to be additive rather than synergistic. Furthermore, Caliskan et al. [38] observed that individuals with high habitual caffeine intake appeared to develop tolerance to this chronotropic effect. In contrast, studies reporting a reduction in HR (3–8 bpm) attributed this finding to the smaller volume of ED administered (250 mL) and to activation of the parasympathetic nervous system. This parasympathetic response may be mediated by baroreceptor stimulation in the carotid arteries secondary to increases in BP or through direct vagal stimulation. However, Costa et al. [39] reported no significant increase in BP in their study, suggesting that parasympathetic activation could not be explained by a baroreceptor-mediated mechanism and may instead be related to other ED constituents, such as taurine.

Several studies have reported alterations in the QTc interval following ED consumption. Some studies observed QTc prolongation [26,29,30,35], whereas others reported QTc shortening [27,29,38]. The EDUCATE study found no significant change in QTc, possibly due to the relatively small amount of ED administered, as previously suggested [31]. In the study by Alsunni et al. [35], QTc prolongation was observed only among overweight or obese male participants, suggesting a potential synergistic effect between ED consumption and elevated BMI. Interestingly, available evidence indicates that QTc alterations may result from the combined effects of multiple ED components rather than from any single ingredient. In the study by Basrai et al. [29], QTc prolongation occurred after consumption of a commercially available ED, whereas QTc shortening was observed after ingestion of control beverages containing taurine with caffeine and glucuronolactone, respectively—an observation reported for the first time in the literature. Notably, consumption of taurine or caffeine alone did not produce similar effects. The reported QTc changes ranged from approximately 6 to 20 ms, although QTc duration remained within normal limits in all the studies mentioned above. Nevertheless, these findings raise concern, as individuals with congenital or acquired QTc prolongation may be at increased risk of developing arrhythmias following ED consumption, a risk also suggested by clinical studies in adults [51]. In 2016, Hajsadeghi et al. [36] reported that more frequent ST-T interval changes were observed after ED consumption, which may indicate subendocardial damage caused by the released catecholamines, while in the study that exclusively involved adolescents [31] an increased incidence of SVES was observed after ED consumption. These specific findings have not been consistently reported in other studies.

The composition of EDs may play a significant role in the development of arrhythmias and electrocardiographic alterations, indicating that these effects cannot be attributed solely to caffeine and taurine, which have been extensively studied in both human and animal models [52,53]. Other constituents may also contribute to these cardiovascular effects but remain insufficiently investigated. Certain ingredients, such as yerba mate and guarana, further increase the total caffeine content of these beverages [2,53]. Panax ginseng was evaluated in one study and demonstrated no significant effects in the studied population [26], whereas another investigation examined a glucuronolactone-containing beverage and reported QTc shortening, as previously mentioned [29]. Although some individual ingredients appear to be relatively harmless when consumed in isolation, further research is required to clarify both their independent effects and, more importantly, their potential synergistic interactions. Such interactions may underline the observed effects of EDs on cardiac rhythm.

Enhanced automaticity, increased supraventricular ectopic activity, and triggered activity mediated by delayed depolarizations are the principal electrophysiological mechanisms that may precipitate AF and, particularly in susceptible individuals, malignant ventricular arrhythmias. Caffeine, especially at high doses, increases sympathetic activation by antagonizing adenosine receptors, leading to elevated circulating catecholamine levels, while at higher concentrations it may also inhibit phosphodiesterase, promote intracellular calcium overload and facilitate triggered activity [54]. Nevertheless, current evidence suggests that these arrhythmogenic effects cannot be attributed to caffeine alone. Other constituents of EDs, including taurine, guarana, sugars, and other stimulants, as well as their combined or synergistic effects, are likely to contribute to the observed electrophysiological alterations. Although none of the controlled trials demonstrated that ED consumption causes severe arrhythmias, consuming large quantities of EDs within a short period particularly in combination with alcohol or illicit drugs, may act as a trigger for these arrhythmias, as suggested by case reports.

The quantity of ED consumed appears to be an important determinant of cardiovascular effects. Studies administering relatively low amounts (e.g., 250 mL of ED or 3 mg/kg of caffeine) have reported a decrease in HR without significant changes in the QTc interval [31,32,34,36,39], in contrast to studies in which larger volumes were administered [26,27,29,30,33,37,38,49]. Furthermore, arrhythmias described in case reports are typically observed following the consumption of relatively high volumes of EDs (approximately 600–750 mL) [42,43,44,45]. In the study by Basrai et al. [29], which evaluated the effects of two different administered volumes (750 mL and 1000 mL), the authors concluded that the observed effects were independent of the administered volume. However, because both volumes were substantially higher than those used in most other studies, this conclusion may not be generalizable.

Additional factors beyond the ED itself may influence cardiovascular responses to ED consumption. The study by Alsunni et al. [35] reported a synergistic effect between ED intake and elevated body mass index (BMI) on HR in young males, whereas Grasser et al. [25] demonstrated an additive effect of mental stress and ED consumption on HR. In 2021, Caliskan et al. [38] suggested that individuals with high habitual caffeine intake may develop tolerance to the cardiovascular effects of caffeine and ED. Case reports further indicate that the combined consumption of EDs with alcohol or recreational drugs may precipitate severe arrhythmias [41,46]. The arrhythmogenic potential of concurrent alcohol and ED consumption has been supported by recent studies [55,56]. In addition, pre-existing cardiovascular conditions and intense physical activity may increase susceptibility to arrhythmic events [42,46]. Although no significant findings raised from the patients’ evaluation, the retrospective nature of case reports limited the ability to account for additional confounding factors that may have contributed to the development of arrhythmias. In one case [46] a variety of unknown significance of MYOMI gene was found, a seemingly not important finding—however, the possibility of genetically predisposed individuals should always be considered. Clinical evidence from the included studies suggests that individuals with pre-existing cardiovascular disease may face an increased risk of arrhythmias due to ED-induced alterations in HR and increased QTc variability. Furthermore, the study by Bezna et al. [40] found that most participants who reported a history of arrhythmia had multiple coexisting risk factors in addition to frequent ED consumption. Therefore, several potentially significant factors, including habitual caffeine intake, BMI, physical activity, dietary patterns, psychological stress, concomitant use of alcohol or other substances, and pre-existing cardiovascular disease, have been identified, but remain insufficiently investigated in the context of ED-related cardiovascular effects. 

An important concern is whether ED consumption is ultimately safe particularly for adolescents. Although all included studies indicate that ED effects on the cardiac electrical activity seem transient and mild to moderate, adolescents are frequently exposed to stressors such as academic examinations, intense physical activity and emotional distress. In addition, risky behaviors such as alcohol consumption, illicit drug use and smoking are common in this age group [20]. Therefore, as mentioned previously, exposure to multiple factors that may act synergistically or additively to affect the cardiovascular responses to ED consumption, likely makes adolescents a high-risk group for adverse effects. As previously mentioned, HR appears to respond differently to ED consumption in younger versus older individuals, showing a decrease in the former and an increase in the latter [31]. One possible explanation for the apparent desensitization observed in young adults is their more frequent consumption of EDs. Compared with adolescents, young adults generally have greater financial independence and fewer restrictions on purchasing these beverages, potentially resulting in higher habitual intake. Available data suggest that habitual caffeine consumption appears to attenuate the effects of EDs, likely through the development of tolerance [38]. However, an interesting question yet to be answered is whether other ED ingredients may also contribute to desensitization through distinct molecular mechanisms.

It is remarkable that the FDA has not established specific regulatory limits for the composition of EDs. In particular, no upper limit for caffeine content or age restrictions for purchase and consumption have been defined, as these products are often classified as dietary supplements [57,58]. In contrast, within the European Union, production companies are required to indicate on the packaging that beverage contains a high amount of caffeine, to specify the amount of caffeine per 100 mL, and to state that the product is not recommended for children, pregnant and breastfeeding women [59]. Some countries have established their own rules such as prohibiting their sale to minors (e.g., Turkey, Latvia, Lithuania) [20].

In 2019, based on data from case studies reporting serious cardiovascular events and clinical trials mainly in adults, the European Cardiac Arrhythmia Society issued recommendations on the consumption of ED, further emphasizing the need for more and larger prospective studies and legislation by the European Union. According to the recommendations:

- EDs should not be consumed by children under 14 years of age or children with underlying heart disease
- Screening should be performed when there is evidence of structural heart disease or arrhythmogenic syndrome (Brugada syndrome, long QT syndrome) as these individuals are at increased risk of sudden cardiac death.
- Daily caffeine consumption in young adults should not exceed 400 mg/day and in children over 14 years of age, daily caffeine consumption should not exceed 2.5 mg/kg of body weight.
- EDs should not be consumed in combination with alcohol or illegal substances and not before sports or intense physical activity [58].

## 5. Limitations and Strengths

Interpretation of the existing evidence should be approached with caution due to several methodological constraints. Many studies involved small sample sizes and primarily included healthy volunteers, with individuals who had underlying medical conditions largely underrepresented. Participant selection was frequently restricted to specific subgroups, such as only males or females, students from a single educational setting, or individuals with normal BMI, which limits the generalizability of the findings. Finally, incomplete reporting of the type and composition of the ED consumed in some studies further complicates comparison and interpretation of the results.

The present review provides a more comprehensive evaluation of the potential arrhythmogenic effects of EDs in their primary consumer groups—adolescents and young adults—based on the most recent literature. Case reports illustrate the “worst-case scenarios” demonstrating that ED consumption may precipitate severe arrhythmias. RCTs, although they may not capture the full extent of possible adverse effects, minimize confounding factors and therefore provide stronger evidence for a causal relationship between ED consumption and ECG alterations, suggesting an increased risk of arrhythmias, particularly in susceptible individuals. Furthermore, although several included studies were non-randomized and lacked blinding of participants and/or outcome assessors—thereby introducing potential bias—their design enabled the investigation of specific variables such as BMI, habitual caffeine consumption and comparisons with other caffeinated beverages.

## 6. Conclusions

The above data show a consistent pattern of findings across the reviewed studies. ED consumption appears to affect the cardiac electrical activity, with the most commonly reported findings being alterations of HR and QTc interval. Although these effects generally appear to be mild to moderate in magnitude and transient in nature, they may have clinically significant implications, particularly in susceptible individuals. Further research involving well-designed clinical studies with larger sample sizes, is warranted to better characterize the long term cardiovascular effects of ED consumption, the individual and combined effects of their various ingredients, and the interactions between EDs, alcohol and other substances. Given the concerning findings reported to date, stricter regulatory measures regarding the marketing, sale and consumption of EDs by minors should be considered, alongside improved public health education on the potential risks associated with excessive or uncontrolled ED consumption, especially among individuals vulnerable to arrhythmias.

## Figures and Tables

**Table 1:** RCTs.

	Reference	Country	N	Age (Median)	Gender (M)	Method/ED/ Quantity	Outcome Measures	Findings
1	Grasser et al. (2015) [25]	Switzerland	20	22.1	50%	355 mL RedBull or 355 mL water in random order + mental arithmetic task	BP, HR, CBFV before and 80 min after drink consumption + mental arithmetic task	RedBull caused SBP, DBP and HR (7 bpm) increase. Combined with mental stress there was a further increase in SBP (10 mmHg), DBP (7 mmHg) and HR (20 bpm) and a decrease in CBFV (−7 cm/s)
2	Shah et al. (2016) [26]	USA	27	21.6	74%	32 οz (~950 mL) ED or Panax Ginseng drink or placebo drink in random order	QTc, SBP, DBP, PR, QRS, QT, HR before, 1, 2, 3^1/2^ and 5^1/2^ h after drink consumption	Significant increase in QTc interval (>6 ms) compared to placebo and in SBP 2 h after consuming the ED. Panax Ginseng caused no significant changes
3	Garcia et al. (2017) [27]	Colombia	80	21	62.50%	460 mL: 1st group (control)—carbonated water, 2nd group—ED A, 3rd group ED B and 4th group ED C. EDs with similar caffeine concentrations but varying sugar, taurine, and other undetermined components.	SBP, DBP, ECG parameters, HR, SpO2, RR, T, anxiety state (STAI score), salivary cortisol, cognitive enhancement (N-back score), before, 30 min and 60 min after drink consumption	SBP increased significantly in groups A and C at 30 min. Increase of DBP and QTc shortening at 60 min in group B. HR showed an increase in the percent change in the A and C groups. Cortisol salivary levels increased in the B group. The STAI test score decreased in the C group. The percentage change in N-back scores increased in the A group.
4	Niemczyk et al. (2017) [28]	Poland	18	23.7	61%	500 mL of ED or placebo in random order	Peripheral and central SBP and DBP, ECG recording, echocardiography and pulse wave velocity analysis	Significant increase of SBP in 75 min of observation compared to placebo. No significant differences in ECG parameters
5	Basrai et al. (2019) [29]	Germany	38	22.3	50%	Group A 750 mL and Group Β 1000 mL of: ED (RedBull) or Control product (CP) or CP + Taurine (Τ) or CP + caffeine (C) in a random order. Group Β also received CP + glucuronolactone (G) and CP + T + C	glucose, insulin, HOMA -IR, caffeine, BP, ECG (QTc, HR), symptoms, before, 1, 3, 7 and 11 h after drink consumption	Changes not volume—depended. Significant SBP, MBP and HR increase (ΔHR = 3.5 bpm) and QTc prolongation (7.5 ± 16.7 ms) 1 h after the ED consumption. CP + C also caused SBP increase. 1 h after CP + G and after CP + T + C consumption a QTc shortening (−9.1 ± 19.0 and −8.7 ± 15.6) was noted. All study products caused a decrease in serum glucose and an increase in insulin concentrations after 1 h compared to baseline values, corresponding to an elevation in the HOMA-IR
6	Shah. et al. (2019) [30]	USA	34	22.1	50%	32 οz (~950 mL) ED A or ED Β or Placebo in random order	QTc, QT, PR, HR, QRS, BP central BP before and every 30 min for 4 h after drink consumption	Significant QTc prolongation (A: +17.9 ± 13.9, B: +19.6 ± 15.8, compared to placebo: +11.9 ± 11.1 ms) and BP increase after consuming EDs A and B. Both EDs had similar effects (similar composition).
7	Mandilaras et al. (2022) [31]	Germany	26	14.5	50%	ED 3 mg of caffeine/kg or placebo drink—in random order	HR, QTc, number of SVES and VES before, 1, 2, 3 and 4 h after drink consumption	Significantly increased number of SVES (IRR: 1.7) and HR significantly lower 60–120 min following consumption of ED compared to placebo (mean difference −2.71 bpm)
8	Oberhoffer et al. (2022) [32]	Germany	27	14.5	52%	ED 3 mg of caffeine/kg or placebo drink—in random order	SBP, DPB, HR (ECG) before, 1, 2, 3 and 4 h after drink consumption	SBP and DBP increase and HR decrease (−2.44 ± 11.10 bpm) after ED consumption
9	Caliskan et al. (2024) [33]	Turkey	56	20.1	71%	4 groups (4 F, 10 M): group 1 (caffeine 200 mg), group 2 (taurine 2500 mg), group 3 (caffeine 200 mg + taurine 2500 mg), control (water)	SBP, DBP, HR (ECG) HRV parameters, before, 30 min and 60 min after drink consumption	SBP and HR increase in C and C + Τ groups (C > C + T). Significant increase in HR in C group at 60 min (89.57 ± 2.06 compared to 79.50 ± 2.11 bpm)
10	Mandilaras et al. (2025) [34]	Germany	26	14.5	50%	ED 3 mg of caffeine/kg or placebo drink—in random order	HR, HRV parameters—Holter ECG, before, 30 min and 60 min after drink consumption	HR decrease 2 h after ED consumption (−2.71 bpm)

ED: energy drink, CBFV: cerebral blood flow velocity, HR: heart rate, BP: blood pressure, SBP: systolic blood pressure, DBP: diastolic blood pressure, STAI: State-Trait Anxiety Inventory, RR: respiratory rate, T: temperature, ECG: electrocardiogram, HOMA-IR: Homeostatic Model Assessment of Insulin Resistance, SVES: supraventricular extrasystoles, VES: ventricular extrasystoles, IRR: incidence rate ratio, HRV: heart rate variability.

**Table 2:** Non-RCTs.

	Reference	Country	N	Age (Median)	Gender (M)	Method/ED/ Quantity	Outcome Measures	Findings
1	Alsunni et al. (2015) [35]	Saudi Arabia	31	20.5	100% (13-NW, 18 OW/OB)	5 mL/kg of an ED (1.6 mg caffeine/kg)	HRV and QTc before, 30 min and 60 min after ED consumption	HRV parameters significantly reduced by ED consumption at 60 min and significant QTc increase at 60 min in the OW/OB group (356.8 ± 54 ms compared to 340.2 ± 57 ms at resting state)
2	Hajsadeghi et al. (2016) [36]	Iran	44	22.5	54%	250 mL of ED	BP, HR, PR, QRS, QT (QTc), ST-T before, 30 min, 2 h and 4 h after ED consumption	Statistically significant HR decrease (ΔHRmean 0–30 min: −3.9 bpm, 0–2 h: −5.1 bpm, 0–4 h: −4.7 bpm) and more frequent ST-T changes
3	Caliskan et al. (2020) [37]	Turkey	48	19.2	58%	473 mL—1st group (12): coffee, 2nd group (12): ED (RedBull), 3rd group (12): Cola, 4th group (12-control): water	HRV and HR before, 30 min and 60 min after drink consumption	Statistically significant HR increase (87.96 ± 3.35 compared to 83.16 ± 2.39 bpm) and increase in nonlinear HRV parameters in the ED group at 60 min
4	Caliskan et al. (2021) [38]	Turkey	48	19.5	67%	Groups of 12 people: Control, ED1 (caffeine naive), ED2 (low-habitual caffeine consumers < 200 mg/day), ED3 (high-habitual caffeine consumers ≥ 200 mg/day)—groups ED 473 mL EΠ (RedBull)	SBP, DBP, RR, ECG (PR, QRS, QTc), SpO2, BIA, stress (STAI) before, 30 and 60 min after ED consumption	SBP, DPB and HR increase and QTc decrease in ED1 and ΕD2 groups (statistically significant HR increase in ED1 87.96 ± 3.54 bpm at 60 min compared to 82.25 ± 2.75 bpm and QTc decrease 0.331 ± 0.007 at 60 min compared to 0.340 ± 0.006) Body metabolism rate increased after ED consumption. No significant changes in ED3 group
5	Costa et al. (2023) [39]	Portugal	30	20	0%	250 mL RedBull	SBP, DBP, HR (ECG), PSV and EDV of carotid arteries and middle cerebral artery, before, 30 min and 60 min after ED consumption	Statistically significant HR decrease, (74.33 ± 2.19 at 60 min compared to 81.47 ± 2.48 bpm). No significant BP changes. Reduction in PSV and EDV.

ED: energy drink, NW: normal weight, OW/OB: overweight/obese, HRV: heart rate variability, BP: blood pressure, SBP: systolic blood pressure, DBP: diastolic blood pressure, HR: heart rate, ΔHR: heart rate difference, RR: respiratory rate, ECG: electrocardiogram, BIA: bioelectrical impedance analysis, STAI: State-Trait Anxiety Inventory, PSV: peak systolic velocity, EDV: endodiastolic velocity.

**Table 3:** Observational study (Cross sectional).

	Reference	Country	N	Age (Median)	Gender (M)	Methods	Outcome Measures	Findings
1	Cristina Beznă et al. (2015) [40]	Romania	186	20	31%	questionnaires	presence or absence of cardiac dysrhythmias, existence of proarrhythmogenic risk factors and determination of oxidative stress status modifications	39% presented heart rhythm disturbances (SVES, sinus tachycardia, VES, paroxysmal AF, paroxysmal SVT, atrial flutter and sinus bradycardia). 72% of those with cardiac rhythm disorders mention ED consumption—31% of those without (*p* < 0.01). Other risk factors present: Coffee consumption 82%, stress 80%, physical effort 72%, hyper lipidic diet 69%, familial predisposition 69%, alcohol intake 53%, frequent sleep deprivation 50%, smoking 31%, OW 31%

ED: energy drink, OW: overweight, SVES: supraventricular extrasystoles, AF: atrial fibrillation, VES: ventricular extrasystoles, SVT: supraventricular tachycardia.

**Table 4:** Case reports.

	Reference	Country	Age/Gender	Medical History	Type of ED/Quantity	Symptoms/Presentation	ECG Findings	Treatment	Results
1	Okutucu et al. (2015) [41]	Turkey	24/M	No	RedBull + Vodka/2 glasses	palpitations	AF with rapid ventricular response	amiodarone	SR after 4 h—further evaluation was normal
2	Enriquez et al. (2017) [42]	USA	19/M	No	Monster/3 cans (~700 mL) in 2 h	cardiac arrest	VF	CPR, defibrillation	S-ICD implantation—further evaluation was normal
			23/F	Peripartum cardiomyopathy S-ICD	RedBull/1 can	Syncope	VF	ICD defibrillation	Recovered
3	Mattioli et al. (2018) [43]	Italy	22/M	No	ED/750 mL	precordial oppressive sensation, palpitations, anxiety, nausea	AF	propafenone	SR—further evaluation was normal
			23/F	No	ED/600 mL	palpitations, stress	AF	spontaneous conversion to SR	SR—further evaluation was normal
4	Hanif et al. (2020) [44]	Pakistan	22/M	No	ΕD/2 cans	shortness of breath, restlessness, vomit	AF with rapid ventricular response	spontaneous conversion to SR	SR—further evaluation was normal
5	Zorlu et al. (2020) [45]	Turkey	23/F	No	ED/750 mL	dyspnea, palpitations, and anxiety	AF	propafenone	SR—further evaluation was normal
6	Dodulik et al. (2024) [46]	Czech Republic	18/M	No	ED + kratom/250 mL + 2 g	cardiac arrest during football training	VF	CPR, defibrillation	ICD implantation—further evaluation was normal (variant of unknown significance found in the MYOM1 gene)

ED: energy drink, AF: atrial fibrillation, SR: sinus rhythm, S-ICD: subcutaneous- implantable cardioverter-defibrillator, VF: ventricular fibrillation, CPR: cardiopulmonary resuscitation.

## Data Availability

The original contributions presented in this study are included in the article. Further inquiries can be directed to the corresponding author.

## Abbreviations

The following abbreviations are used in this manuscript:

AF	Atrial Fibrillation
BIA	Bioelectrical Impedance Analysis
BP	Blood Pressure
CPR	cardiopulmonary resuscitation
CBFV	Cerebral Blood Flow Velocity
DBP	Diastolic Blood Pressure
ECG	Electrocardiogram
ED	Energy Drinks
EDV	Endo-diastolic Velocity
FDA	Food and Drug Administration
HOMA-IR	Homeostatic Model Assessment of Insulin Resistance
HR	Heart Rate
HRV	Heart Rate Variability
Non-RCT	Non-Randomized Controlled Trial
NW	normal weight
OW/OB	Overweight/obese
PSV	Peak Systolic Velocity
RCT	Randomized Controlled Trial
RR	Respiratory Rate
S-ICD	subcutaneous- implantable cardioverter-defibrillator
SANRA	Scale for the Assessment of Narrative Review Articles
SBP	Systolic Blood Pressure
SR	Sinus Rhythm
STAI	State-trait anxiety index
SVES	Supraventricular Extrasystoles
VES	Ventricular Extrasystoles
VF	Ventricular Fibrillation

## Appendix A. SANRA—Scale for the Assessment of Narrative Review Articles

(1) Justification of the article’s importance for the readership
  The importance is not justified (0)
  The importance is alluded to, but not explicitly justified (1)
  The importance is explicitly justified (2)
(2) Statement of concrete aims or formulation of questions
  No aims or questions are formulated (0)
  Aims are formulated generally but not concretely or in terms of clear questions (1)
  One or more concrete aims or questions are formulated (2)
(3) Description of the literature search
  The search strategy is not presented (0)
  The literature search is described briefly (1)
  The literature search is described in detail, including search items and inclusion criteria (2)
(4) Referencing
  Key statements are not supported by references (0)
  The referencing of key statements is inconsistent (1)
  Key statements are supported by references (2)
(5) Scientific reasoning
  The article’s point is not based on appropriate arguments (0)
  Appropriate evidence is introduced selectively (1)
  Appropriate evidence is generally present (2)
(6) Appropriate presentation of data
  Data are presented inadequately (0)
  Data are often not presented in the most appropriate way (1)
  Relevant outcome data are generally presented appropriately (2)

**Table A1:** SANRA score.

Domain	Score (0–2)
Justification of the article’s importance for the readership	2
Statement of concrete aims or formulation of questions	2
Description of the literature search	2
Referencing	2
Scientific reasoning	2
Appropriate presentation of data	2
Total score	12
